# Blood Levels of Organochlorine Contaminants Mixtures and Cardiovascular Disease

**DOI:** 10.1001/jamanetworkopen.2023.33347

**Published:** 2023-09-12

**Authors:** Carolina Donat-Vargas, Tessa Schillemans, Hannu Kiviranta, Panu Rantakokko, Ulf de Faire, Juan Pedro Arrebola, Alicja Wolk, Karin Leander, Agneta Åkesson

**Affiliations:** 1 Cardiovascular and Nutritional Epidemiology, Institute of Environmental Medicine, Karolinska Institutet, Stockholm, Sweden; 2 ISGlobal, Barcelona, Spain; 3 CIBER Epidemiología y Salud Pública, Madrid, Spain; 4 Department of Health Security, National Institute for Health and Welfare, Kuopio, Finland; 5 Universidad de Granada, Department of Preventive Medicine and Public Health, Granada, Spain; 6 Instituto de Investigación Biosanitaria, Granada, Spain

## Abstract

**Importance:**

Cardiovascular toxic effects derived from high exposures to individual organochlorine compounds are well documented. However, there is no evidence on low but continuous exposure to combined organochlorine compounds in the general population.

**Objective:**

To evaluate the association of combined exposure to several organochlorine compounds, including organochlorine pesticides and polychlorinated biphenyls, with incident cardiovascular disease (CVD) in the general population.

**Design, Setting, and Participants:**

This prospective nested case–control study included data from 2 cohorts: the Swedish Mammography Cohort-Clinical (SMC-C) and the Cohort of 60-Year-Olds (60YO), with matched case-control pairs based on age, sex, and sample date. Baseline blood sampling occurred from November 2003 to September 2009 (SMC-C) and from August 1997 to March 1999 (60YO), with follow-up through December 2017 (SMC-C) and December 2014 (60YO). Participants with myocardial infarction or ischemic stroke were matched with controls for composite CVD evaluation. Data were analyzed from September 2020 to May 2023.

**Exposures:**

A total of 25 organochlorine compounds were measured in blood at baseline by gas chromatography–triple quadrupole mass spectrometry. For 7 compounds, more than 75% of the samples were lower than the limit of detection and not included.

**Main Outcomes and Measures:**

Incident cases of primary myocardial infarction and ischemic stroke were ascertained via linkage to the National Patient Register (*International Statistical Classification of Diseases and Related Health Problems, Tenth Revision* codes I21 and I63). The quantile-based g-computation method was used to estimate the association between the combined exposure to several organochlorine compounds and composite CVD.

**Results:**

Of 1528 included participants, 1024 (67.0%) were female, and the mean (SD) age was 72 (7.0) years in the SMC-C and 61 (0.1) years in the 60YO. The odds ratio of composite CVD was 1.71 (95% CI, 1.11-2.64) per 1-quartile increment of total organochlorine compounds mixture. Organochlorinated pesticides were the largest contributors, and β-hexachlorocyclohexane and transnonachlor had the highest impact. Most of the outcome was not explained by disturbances in the main cardiometabolic risk factors, ie, high body mass index, hypertension, lipid alteration, or diabetes.

**Conclusions and Relevance:**

In this prospective nested case-control study, participants with higher exposures to organochlorines had an increased probability of experiencing a cardiovascular event, the major cause of death worldwide. Measures may be required to reduce these exposures.

## Key Points

**Question:**

Can persistent organochlorine compounds cause cardiovascular disease (CVD) in general populations exposed at low doses via the diet?

**Findings:**

In this nested case-control study including 345 participants with myocardial infarction, 354 participants with ischemic stroke, and 829 controls, biomarkers reflecting exposure to organochlorines exhibited a significant association with heightened odds of composite CVD, encompassing myocardial infarction and stroke. Per each quartile increment in the total compound mixture, the likelihood of experiencing a cardiovascular event increased significantly by 71%.

**Meaning:**

This study suggests a significant and independent role of organochlorine compounds exposure in the pathogenesis of CVD.

## Teaser

This nested case-control study evaluates the association of combined exposure to several organochlorine compounds, including organochlorine pesticides and polychlorinated biphenyls, with incident cardiovascular disease in the general population.

## Introduction

Persistent organochlorine compounds (OCs), including organochlorinated pesticides (OCPs), dioxins, and polychlorinated biphenyls (PCBs), are toxic synthetic lipophilic chemicals that bioaccumulate in the fatty tissue of living organisms. Despite their regulation and legislation,^1^ they remain prevalent in our environment, and upon exposure, mainly through consumption of animal fat,^2,3,4^ OCs accumulate in humans worldwide.^5^

Cardiovascular disease (CVD) is the primary global cause of premature mortality, with ischemic heart disease (including myocardial infarction [MI]) being the first and cerebrovascular diseases the third contributor to years of life lost.^6,7,8^ Identification of environmental risk factors is important for public health, as a widespread exposure together with a high incidence means that risks of seemingly minor magnitude can exert population-level impacts. Experimental and animal studies have shown that PCB exposure may impair endothelial function and induce atherosclerosis via oxidative stress, inflammation, immunotoxicity-related mechanisms, and endocrine disruption^9^ or may even alter gene expression patterns in vascular cells.^10^ In humans, an association between OCs and CVD was initially suggested by industrial accidents, high occupational exposure,^11,12^ and ecological studies in individuals living close to contaminated waste sites.^13^ These findings were followed by associations observed in the general population for both subclinical coronary atherosclerosis^14^ and the major CVD risk factors, such as hypertension,^15^ dyslipidemia,^16^ diabetes,^17^ and obesity.^18^ Diet-derived OC exposure has been associated with several CVD outcomes,^14,19,20,21,22^ but studies using biomarkers of exposure are scarce. While elevated blood concentrations of both PCBs and OCPs have been associated with incident stroke in Nordic^23^ and Korean^24^ populations, the association with incident MI is unexplored, as is the impact on these end points of the joint toxic effect of several compounds. Integrating multiple chemical exposures provides a more realistic estimate of risk than exposures to single specific compounds and is more meaningful for health risk assessment, public health prevention, or interventions.^25,26^

Herein, we used an improved method for environmental chemical mixtures within epidemiological studies to account for the cooccurrence of multiple components present in the real world as a complex mixture.^27^ Thus, using quantile g-computation, we examined the joint mixture association of the most prevalent OCPs and PCBs congeners with the incidences of MI and ischemic stroke in 2 population-based cohorts, using a prospective nested case-control design.

## Methods

Written or oral informed consent was obtained from all participants in the original cohorts, and the studies were approved by the regional ethical review board in Stockholm. This study followed the Strengthening the Reporting of Observational Studies in Epidemiology (STROBE) reporting guideline.

### Study Population and Ascertainment of Cardiovascular Outcomes

The study used data from the Swedish Mammography Cohort-Clinical (SMC-C)^28^ and the Cohort of 60-Year-Olds (60YO).^29^ The SMC, established between 1987 and 1990, included women born from 1914 to 1948 residing in central Sweden (74% response rate; n = 61 433).^30^ Between November 2003 and September 2009, all SMC-women younger than 85 years living in Uppsala and surrounding areas were invited for a health examination (used as baseline in this study); 5022 responders (61% response rate) constituted the SMC-C. The 60YO, established to investigate CVD etiology, identified residents in Stockholm County turning 60 years old between July 1997 and June 1998 and randomly invited every third man and woman for a health examination between August 1997 and March 1999 (78% response rate; n = 4232). Both cohorts donated blood samples and completed a questionnaire.^31^

From baseline blood sampling (2003 to 2009 in SMC-C and 1997 to 1999 in 60YO) in CVD-free participants, a total of 135 and 214 incident cases of MI, respectively, and 173 and 183 incident cases of ischemic stroke were ascertained via linkage to the National Patient Register (*International Statistical Classification of Diseases and Related Health Problems, Tenth Revision* codes I21 and I63) through December 2017 for the SMC-C and December 2014 for the 60YO. The included outside hospital deaths from MI from the Cause of Death Register were verified by autopsy reports. Diagnosis of MI and stroke were correct in 98.0% and 98.6% of the individuals, respectively, in a validation study.^32^

For each case, controls were randomly matched if they were alive and free from the case diagnosis (MI or stroke) at the time the case experienced the event (risk-set sampling). In the SMC-C, controls were matched (1:2 for MI and 1:1 for stroke) based on age (within 1 year) and sample date (within 90 days). In the 60YO, controls were matched (1:1 for both MI and stroke) based on sex and sample date (within 90 days). Due to some missing samples (broken vials corresponding to 6 cases), the final study population constituted of 134 cases and 264 controls in the SMC-C and 211 case-control pairs in the 60YO for MI assessment and 172 pairs in the SMC-C and 182 pairs in the 60YO for ischemic stroke assessment. Thus, 699 cases and 829 controls were available for composite CVD evaluation (eFigure 1 in Supplement 1).

### Baseline Measurements of OCs and Individual Data Collection

Blood samples were collected after an 8-hour overnight fasting and were immediately centrifuged, separated, and stored at −80 °C. A total of 25 compounds were measured in plasma, of which 12 PCBs (congeners 52, 74, 99, 101, 118, 138, 153, 156, 170, 180, 183, and 187) and 6 OCPs or their metabolites (dichlorodiphenyltrichloroethane, dichlorodiphenyldichloroethene [p,p′-DDE], β-hexachlorocyclohexane [β-HCH], hexachlorobenzene [HCB], transnonachlor, and oxychlordane were included, while 7 were not considered because more than 75% of the samples were below the limit of quantification). For comprehensive details, see the eMethods and eTable 1 in Supplement 1.

### Baseline Assessment of Covariates

Self-reported questionnaire information included age, sex, education, comorbidities, family history of MI (ie, heart attack in a first-degree relative younger than 60 years), smoking, and physical activity (participants were categorized as physically active when reporting walking and/or biking for 40 minutes or more per day and exercising 1 hour or more per week in the SMC-C and when they categorized their own level of activity as moderate or high in the 60YO). Height and weight were measured to calculate body mass index (BMI; calculated as weight in kilograms divided by height in meters squared).

Dietary information was obtained from a semiquantitative 124-item food frequency questionnaire in the SMC-C and from a questionnaire with a total of 17 food-related questions in the 60YO. For the SMC-C, we created a healthy diet score based on the 8-point scoring system (low to high adherence) of the modified Mediterranean diet score^33^ and collapsed into 3 categories (unhealthy, moderately healthy, or healthy). For the 60YO, the healthy diet score was constructed from a 6-point scoring system based on intakes of fruits, vegetables, fish, and alcohol (in moderation) as positive components and with meat and snacks as negative components and was also collapsed into the same 3 categories. Blood lipids were measured in blood plasma (SMC-C) or serum (60YO) after overnight fasting using routine hospital laboratories in the SMC-C and automated measurement systems in the 60YO.

### Statistical Analysis

Pairwise correlations were assessed using the Spearman rank correlation coefficient (ρ). We analyzed cohort-specific tertiles and quartiles (based on the concentration among controls) of individual and grouped OCs (ie, OCPs, dioxinlike [DL] PCBs, non-DL PCBs, and total PCBs), respectively. For OC grouping details, see the eMethods in Supplement 1.

Odds ratios (ORs) and 95% CIs for composite CVD, MI, and ischemic stroke were estimated using conditional logistic regressions in both cohorts pooled and in each cohort separately. To test for linear trends across increasing categories of exposures, we used the median concentration within each category as a continuous variable.

As the main analysis, we estimated the ORs and 95% CIs of composite CVD per 1-quartile increment of total exposure to OC mixture using the quantile-based g-computation approach (R package qgcomp).^34^ The overall OC mixture effect size from quantile-based g-computation is interpreted as the impact on the outcome (ie, composite CVD) of increasing all exposures at once by 1 quantile conditional on covariates. Covariates were selected based on a priori knowledge of CVD risk and protective factors that could also affect the levels of the OCs.^35,36^ In addition to matching (age in SMC-C, sex in 60YO, and sample year), the covariates included in the regression models were: attained education (12 years or less vs more than 12 years), physical activity (active or inactive), smoking habits (never, former, or current), healthy diet score (unhealthy, moderately healthy, or healthy), fish consumption (tertiles in the SMC-C and 2 categories in the 60YO), BMI (25 or less, more than 25 to 30, or more than 30), and family history of MI (yes or no), which were all measured at baseline. Diabetes, hypertension, and lipid levels were exclusively introduced as adjustments in a supplementary model. While these variables hold the potential to act as confounders, they could also serve as intermediators^18,37^ or potentially introduce collider bias in the association between OCs and CVD.

We created missing indicator variables for confounders with missing values. The proportion of missing data was less than 5% for all covariates, except for physical activity in the SMC-C, which was 16%. Statistical analyses were performed using Stata version 15.1 (StataCorp) and R version 3.6.1 (The R Foundation). All *P* values were 2-tailed, and the level of significance was set at *P* < .05.

## Results

Of 1528 included participants, 1024 (67.0%) were female, and the mean (SD) age was 72 (7.0) years in the SMC-C and 61 (0.1) years in the 60YO (Table 1). Comparing cohorts, the SMC-C encompassed an older population (11 years older, on average) with a later sampling date and a slightly lower prevalence of diabetes and hypertension compared with the 60YO. Comparing controls vs cases in both cohorts, overall, the controls were more educated, less often smokers, had a healthier diet, and had a lower prevalence of diabetes and hypertension and lower triglyceride levels.

**Table 1.** Baseline Characteristics and Concentrations of Organochlorine Compounds (OCs) by Composite Cardiovascular Disease (CVD) Case-Control Status of 742 Women From the Swedish Mammography Cohort-Clinical (SMC-C) and of 786 Men and Women From the Swedish 60-Year-Olds Cohort (60YO)

Characteristic	SMC-C		60YO	
	Cases (n = 306)	Controls (n = 436)	Cases (n = 393)	Controls (n = 393)
**Characteristics, No. (%)**				
Sex				
Female	306 (100)	436 (100)	141 (36)	141 (36)
Male	0	0	252 (64)	252 (64)
Age, mean (SD), y	72 (7.3)	72 (7.3)	61 (0.1)	61 (0.1)
Sample year, mean (SD)	2006 (1.5)	2006 (1.5)	1998 (0.4)	1998 (0.3)
Education ≥12 y	93 (30)	138 (32)	79 (20)	125 (32)
Physically inactive	175 (57)	249 (57)	257 (65)	255 (65)
Smoking status				
Never	167 (55)	266 (61)	135 (35)	188 (47)
Former	92 (30)	133 (31)	135 (34)	144 (37)
Current	47 (15)	37 (8)	123 (31)	61 (16)
Diet score^a^				
Unhealthy	73 (21)	74 (17)	171 (44)	137 (34)
Moderately healthy	180 (62)	256 (59)	130 (33)	124 (32)
Healthy	53 (17)	106 (24)	92 (23)	132 (34)
Fish consumption				
<1.5 Times/wk	109 (36)	142 (33)	NA	NA
1.5 to <3 Times/wk	104 (34)	142 (33)	NA	NA
≥3 Times/wk	90 (29)	146 (33)	NA	NA
Fish consumption				
<1 Times/wk	NA	NA	126 (32)	104 (26)
≥1 Times/wk	NA	NA	243 (62)	280 (71)
Body mass index^b^				
≤25	107 (35)	188 (43)	111 (28)	131 (33)
>25 and ≤30	138 (45)	168 (39)	197 (50)	180 (46)
>30	61 (20)	80 (18)	85 (22)	82 (21)
Family history of CVD	115 (38)	156 (36)	171 (44)	173 (44)
Diabetes	14 (5)	12 (3)	37 (9)	23 (6)
Hypertension	160 (52)	179 (41)	195 (50)	150 (38)
Total cholesterol, mean (SD), mg/dL	224.3 (42.5)	224.3 (38.7)	232.0 (38.7)	228.1 (38.7)
Triglycerides, mean (SD), mg/dL	132.7 (62.0)	115.0 (53.1)	141.6 (88.5)	123.9 (79.7)
**OCs, median (IQR), pg/mL**				
p,p′-DDE	2065.5 (1005-3476)	1732 (921-3098.5)	2516 (1434-4241.5)	2269 (1312.5-3716)
p,p′-DDT	24 (10.5-42)	21 (10.5)-42	46 (26.5-77)	43 (25-77.5)
HCB	175 (134-228)	160 (124.5-220)	233 (181-311)	230 (180.5-298)
β-HCH	124 (80-184)	115.5 (69-178)	182 (123.5-283)	161.5 (103.5-241)
Transnonachlor	84 (61-115)	79 (56.5-109)	97.5 (67.5-146)	91.5 (63.5-124.5)
Oxychlordane	47 (34-62)	41 (31-57.5)	48 (35.5-68)	47 (33.5-61)
PCB-52	7 (3.5-10)	7 (3.5-11)	3.5 (3.5-3.5)	3.5 (3.5-3.5)
PCB-74	57 (44-75)	55 (39-79)	63 (43.5-90.5)	61 (41-83.5)
PCB-99	49 (32-70)	49 (31-73)	70 (46-99)	63 (43-95.5)
PCB-101	5 (3.5-7)	5 (3.5-8)	5 (3.5-8.5)	5 (3.5-8.5)
PCB-118	144 (92-198)	136 (90.5-203)	154.5 (103.5-226-5)	156 (103-231.5)
PCB-138	571.5 (422-766)	558.5 (387.5-765.5)	701.5 (505-948)	641 (462-894.5)
PCB-153	991.5 (756-1239)	951.5 (688-5-1255.5)	1141 (860-1545)	1062.5 (811-14.76)
PCB-156	115 (89-147)	110 (85-141)	132 (100-177)	123 (94-164)
PCB-170	330.5 (267-419)	324 (260-399.5)	402 (303-520.5)	383 (287-483)
PCB-180	734.5 (588-907)	700 (569.5-885.5)	866.5 (646.5-1092)	815.5 (638-1083)
PCB-183	59 (42-80)	57 (39.5-79.5)	78 (52-106.5)	69.5 (50.5-103)
PCB-187	188 (144-240)	177 (133.5-243.5)	220 (158-294)	199.5 (149-279.5)

Abbreviations: HCB, hexachlorobenzene; β-HCH, β-hexachlorocyclohexane; NA, not applicable; p,p′-DDE, dichlorodiphenyldichloroethene; p,p′-DDT, dichlorodiphenyltrichloroethane; PCB, polychlorinated biphenyl.

SI conversion factor: To convert cholesterol to mmol/L, multiply by 0.0259; triglycerides to mmol/L, multiply by 0.0113.

^a^ Diet score is collapsed into the 3 categories (unhealthy, moderately healthy, or healthy) from an 8-point scoring system (low to high adherence) of the modified Mediterranean diet score for the SMC-C and from a 6-point scoring system based on intakes of fruits, vegetables, fish, and alcohol (in moderation) as positive components and with meat and snacks as negative components for the 60YO.

^b^ Calculated as weight in kilograms divided by height in meters squared.

### OC Levels and Correlations

With some exceptions, the mean concentrations of individual OCs were slightly higher in the 60YO than in the SMC-C. Overall, most of the OC concentrations were slightly higher in cases than in controls (Table 1). The highest concentrations of single OCs were p,p′-DDE (IQR, 1135 to 3545 pg/mL), followed by PCB-153 (IQR, 770 to 1379 pg/mL), PCB-180 (IQR, 602 to 993 pg/mL), and PCB-138 (IQR, 432 to 845 pg/mL) (eFigure 2 in Supplement 1).

Several PCBs were strongly correlated with each other (ρ greater than 0.80). The most strongly correlated OCPs pairs were oxychlordane with transnonachlor (ρ = 0.89), HCB with β-HCB (ρ = 0.79), and HCB with oxychlordane (ρ = 0.74). Likewise, transnonachlor was also strongly correlated with PCB-187 (ρ = 0.78). The rest of the compounds were moderately to weakly correlated (eFigure 3 in Supplement 1). Groups of OCs were not highly correlated with lipid levels (eTable 5 in Supplement 1).

### OCPs and CVD

The standardized-based sum of OCPs was associated with 60% higher odds of composite CVD comparing the highest quartile with the lowest (OR, 1.60; 95% CI, 1.14-2.24; *P* for trend = .004) (Table 2). After successively adjusting for potential intermediate factors (blood lipids, hypertension, and diabetes), the ORs were slightly attenuated but remained clinically relevant (eTable 2 in Supplement 1). Equivalent ORs for MI and for ischemic stroke were 1.95 (95% CI, 1.19-3.19; *P* for trend = .08) and 1.55 (95% CI, 0.95-2.51; *P* for trend = .046), respectively (Table 3). Individual OCPs were associated with composite CVD, with ORs ranging from 1.17 (95% CI, 0.87-1.57) for HCB to 1.54 (95% CI, 1.11-2.13) for β- HCH (Table 2). Likewise, associations were maintained in both cohorts separately, with an OR for standardized-based sum of OCP of 1.67 (95% CI, 0.99-2.84; *P* for trend = .09) in the SMC-C and 1.65 (95% CI, 1.04-2.60; *P* for trend = .02) in the 60YO, when highest quartile was compared with the lowest (eTable 3 in Supplement 1).

**Table 2.** Multivariable-Adjusted Prospective Associations Between Plasma Biomarkers of the Total and Single Organochlorine Pesticide (OCP) Exposure and Subsequent Risk of Composite Cardiovascular Disease (CVD) in 1528 Men and Women From the 2 Pooled Cohorts

Measure	Composite CVD			
	Controls, No.	Cases, No.	OR (95% CI)^a^	OR (95% CI)^b^
Total OCPs^c^				
Quartile 1	208	137	1 [Reference]	1 [Reference]
Quartile 2	207	168	1.26 (0.93-1.70)	1.15 (0.83-1.61)
Quartile 3	207	171	1.29 (0.95-1.77)	1.21 (0.86-1.69)
Quartile 4	207	223	1.69 (1.24-2.31)	1.60 (1.14-2.24)
*P* value for trend	NA	NA	.001	.004
Single OCPs				
p,p′-DDE				
Tertile 1	277	201	1 [Reference]	1 [Reference]
Tertile 2	276	218	1.13 (0.87-1.47)	1.03 (0.77-1.36)
Tertile 3	276	280	1.45 (1.12-1.89)	1.22 (0.92-1.62)
*P* value for trend	NA	NA	.004	.13
p,p′-DDT				
Tertile 1	131	121	1 [Reference]	1 [Reference]
Tertile 2	424	316	1.01 (0.74-1.39)	1.19 (0.84-1.68)
Tertile 3	274	262	1.24 (0.90-1.70)	1.46 (1.02-2.07)
*P* value for trend	NA	NA	.07	.04
HCB				
Tertile 1	277	218	1 [Reference]	1 [Reference]
Tertile 2	277	222	1.04 (0.80-1.35)	1.02 (0.77-1.35)
Tertile 3	275	259	1.21 (0.93-1.59)	1.17 (0.87-1.57)
*P* value for trend	NA	NA	.14	.27
β-HCH				
Tertile 1	278	181	1 [Reference]	1 [Reference]
Tertile 2	275	234	1.37 (1.04-1.81)	1.25 (0.92-1.69)
Tertile 3	276	284	1.75 (1.31-2.33)	1.54 (1.11-2.13)
*P* value for trend	NA	NA	<.001	.01
Transnonachlor				
Tertile 1	276	192	1 [Reference]	1 [Reference]
Tertile 2	276	233	1.25 (0.96-1.62)	1.16 (0.88-1.54)
Tertile 3	277	274	1.46 (1.11-1.92)	1.44 (1.08-1.93)
*P* value for trend	NA	NA	.01	.01
Oxychlordane				
Tertile 1	280	200	1 [Reference]	1 [Reference]
Tertile 2	278	233	1.20 (0.91-1.58)	1.13 (0.85-1.52)
Tertile 3	271	266	1.39 (1.06-1.82)	1.32 (0.99-1.76)
*P* value for trend	NA	NA	.02	.06

Abbreviations: HCB, hexachlorobenzene; β-HCH, β-hexachlorocyclohexane; NA, not applicable; OR, odds ratio; p,p′-DDE, dichlorodiphenyldichloroethene; p,p′-DDT, dichlorodiphenyltrichloroethane.

^a^ Model adjusted for matching factors (sex, age, sampling date, and cohort).

^b^ Model adjusted for matching factors (sex, age, sampling date, and cohort), education, physical activity, smoking status, healthy diet score, fish consumption, body mass index, and family history of myocardial infarction.

^c^ Total OCPs include p,p′-DDE, p,p′-DDT, HCB, β-HCH, transnonachlor, and oxychlordane.

**Table 3.** Multivariable-Adjusted Prospective Associations Between Total Plasma Concentrations of Organochlorine Pesticides (OCPs) or Polychlorinated Biphenyls (PCBs) and Subsequent Risk of Myocardial Infarction and Ischemic Stroke in 1528 Men and Women From 2 Pooled Cohorts

Measure	Controls, No.	Cases, No.	OR (95%CI)^a^	OR (95%CI)^b^
**Myocardial infarction**				
Total OCPs^c^				
Quartile 1	119	57	1 [Reference]	1 [Reference]
Quartile 2	119	90	1.60 (1.05-2.44)	1.48 (0.94-2.35)
Quartile 3	119	85	1.54 (0.98-2.41)	1.25 (0.76-2.06)
Quartile 4	118	113	2.09 (1.34-3.25)	1.95 (1.19-3.19)
*P* value for trend	NA	NA	.01	.08
Total PCBs^d^				
Quartile 1	120	68	1 [Reference]	1 [Reference]
Quartile 2	118	90	1.33 (0.89-1.99)	1.27 (0.82-1.97)
Quartile 3	120	105	1.55 (1.03-2.34)	1.31 (0.84-2.06)
Quartile 4	117	82	1.23 (0.79-1.91)	1.15 (0.71-1.85)
*P* value for trend	NA	NA	.18	.52
**Ischemic stroke**				
Total OCPs^c^				
Quartile 1	89	72	1 [Reference]	1 [Reference]
Quartile 2	88	74	1.05 (0.67-1.65)	1.05 (0.64-1.73)
Quartile 3	89	102	1.46 (0.94-2.27)	1.58 (0.98-2.53)
Quartile 4	88	106	1.56 (1.00-2.43)	1.55 (0.95-2.51)
*P* value for trend	NA	NA	.03	.05
Total PCBs^d^				
Quartile 1	89	77	1 [Reference]	1 [Reference]
Quartile 2	88	70	0.92 (0.58-1.44)	0.94 (0.58-1.53)
Quartile 3	89	103	1.35 (0.89-2.06)	1.29 (0.82-2.04)
Quartile 4	88	104	1.41 (0.91-2.19)	1.35 (0.84-2.18)
*P* value for trend	NA	NA	.05	.12

Abbreviations: NA, not applicable; OR, odds ratio.

^a^ Model adjusted for matching factors (sex, age, sampling date, and cohort).

^b^ Model adjusted for matching factors (sex, age, sampling date, and cohort), education, physical activity, smoking status, healthy diet score, fish consumption, body mass index, and family history of myocardial infarction.

^c^ Total OCP include dichlorodiphenyldichloroethene, dichlorodiphenyltrichloroethane, hexachlorobenzene, β-hexachlorocyclohexane, transnonachlor, and oxychlordane.

^d^ Total PCBs include all PCB congeners. Dioxinlike PCBs include PCB-118 and PCB-156; non-dioxinlike PCBs include PCB-28, PCB-52, PCB-74, PCB-99, PCB-10, PCB-138, PCB-153, PCB-170, PCB-180, PCB-183, and PCB-187.

### PCBs and CVD

When the highest quartile was compared with the lowest quartile of total PBC (standardized-based summary of individual PCB congeners), the HR was greater than 1 but not statistically significant (multivariable-adjusted OR, 1.28; 95% CI, 0.92-1.79; *P* for trend = .13) (Table 4). Similar findings were obtained for both DL and non-DL PCBs. These results were attenuated after successively adjusting the models for potential intermediate factors (blood lipids, hypertension, and diabetes) (eTable 2 in Supplement 1). Likewise, ORs for MI and ischemic stroke were not significant (MI: multivariable-adjusted OR, 1.15; 95% CI, 0.71-1.85; *P* for trend = .52; ischemic stroke: multivariable-adjusted OR, 1.35; 95% CI, 0.84-2.18; *P* for trend = .12) (Table 3). As to individual PCB congeners, there were no clear associations with higher odds of composite CVD (eTable 4 in Supplement 1), even in cohort-specific analyses (eTable 3 in Supplement 1).

**Table 4.** Multivariable-Adjusted Prospective Associations Between Biomarkers of the Total and Dioxinlike and Non-Dioxinlike Polychlorinated Biphenyls (PCBs) and Risk of Composite Cardiovascular Disease (CVD) in 1528 Men and Women From 2 Pooled Cohorts

Measure	Controls, No.	Cases, No.	Composite CVD	
			OR (95% CI)^a^	OR (95% CI)^b^
Total PCBs^c^				
Quartile 1	208	143	1 [Reference]	1 [Reference]
Quartile 2	209	161	1.12 (0.83-1.51)	1.11 (0.81-1.52)
Quartile 3	206	208	1.47 (1.09-1.98)	1.35 (0.98-1.86)
Quartile 4	206	187	1.35 (0.99-1.84)	1.28 (0.92-1.79)
*P* value for trend	NA	NA	.04	.13
Dioxinlike PCBs^c^				
Quartile 1	208	150	1 [Reference]	1 [Reference]
Quartile 2	207	174	1.19 (0.89-1.61)	1.13 (0.82-1.55)
Quartile 3	207	190	1.27 (0.94-1.72)	1.27 (0.92-1.76)
Quartile 4	207	185	1.25 (0.91-1.71)	1.27 (0.91-1.78)
*P* value for trend	NA	NA	.21	.15
Non-dioxinlike PCBs^c^				
Quartile 1	208	154	1 [Reference]	1 [Reference]
Quartile 2	207	167	1.12 (0.83-1.49)	1.08 (0.79-1.48)
Quartile 3	207	198	1.32 (0.98-1.78)	1.18 (0.86-1.63)
Quartile 4	207	180	1.17 (0.86-1.59)	1.12 (0.81-1.56)
*P* value for trend	NA	NA	.31	.50

Abbreviations: NA, not applicable; OR, odds ratio.

^a^ Model adjusted for matching factors (sex, age, sampling date, and cohort).

^b^ Model adjusted for matching factors (sex, age, sampling date, and cohort), education, physical activity, smoking status, healthy diet score, fish consumption, body mass index, and family history of myocardial infarction.

^c^ Total PCBs include all PCB congeners. Dioxinlike PCBs include PCB-118 and PCB-156; non-dioxinlike PCBs include PCB-28, PCB-52, PCB-74, PCB-99, PCB-10, PCB-138, PCB-153, PCB-170, PCB-180, PCB-183, and PCB-187.

### Joint OCs Mixture and Composite CVD

The multivariable-adjusted OR for composite CVD was 1.71 (95% CI, 1.11-2.64) per 1-quartile increment of the overall OC mixture (Figure). The OR remained elevated after adjusting for potential intermediate factors (OR per 1-quartile increment, 1.61; 95% CI, 1.01-2.54). The individual OCs with the highest contribution to the mixture effect size were 2 pesticides, β-HCH and transnonachlor, followed by the non-DL PCB-52, PCB-170, and PCB-74 (Figure).

**Figure.**
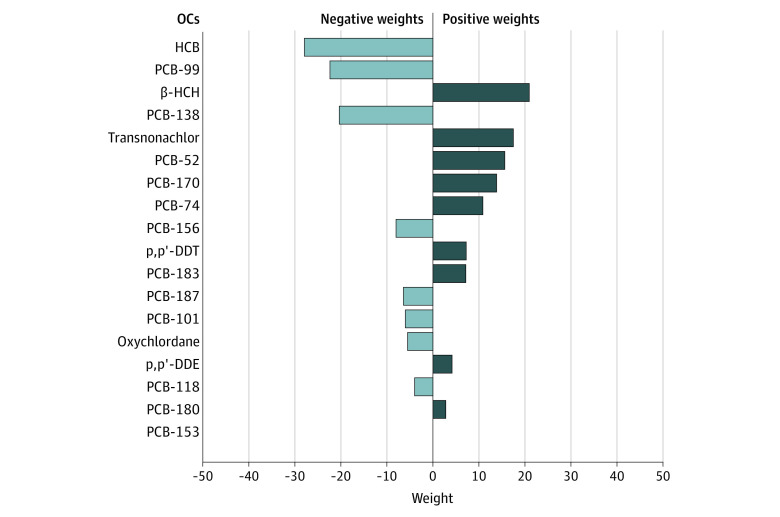
Estimation of the Mixture Effect Size by 1-Quartile Increment in the Biomarkers of Exposure to the Different Organochlorine Compounds (OCs) on Subsequent Risk of Composite Cardiovascular Disease Estimated by Quantile-Based g-Computation Among 829 Controls and 699 Cases Weights for each OC in the mixture effect size. The plot displays the directions and magnitude of the assigned weights (contribution) for each OC within the mixture and its associations with composite cardiovascular disease in quantile g-computation. The weights correspond to the proportion of the effect size in a particular direction. Note that the left and right sides of the plot should not be compared because the length of the bars only corresponds to the effect size relative to the others in the same direction. The darkness of the bars corresponds to the overall effect size, which allows to make informal comparisons across the left and right sides: a large, darker bar indicates a larger independent effect than a large, more lightly shaded bar. The bars on the right (positive) side of the plot are darker because the overall mixture effect size is positive. Model 1 adjusted for matching factors (sex, age, sampling date, and cohort); the odds ratio per 1-quartile increment was 1.96 (1.30-2.95). Model 2 adjusted for matching factors (sex, age, sampling date, and cohort), education, physical activity, smoking status, healthy diet score, fish consumption, body mass index, and family history of myocardial infarction; the odds ratio per 1-quartile increment was 1.71 (95% CI, 1.11-2.64). HCB indicates hexachlorobenzene; β-HCH, β-hexachlorocyclohexane; p,p′-DDE, dichlorodiphenyldichloroethene; p,p′-DDT, dichlorodiphenyltrichloroethane.

## Discussion

To our knowledge, this is the most comprehensive study on the association between biomarkers of exposure to OCs and incident cardiovascular events in the general population to date. Our investigation yielded compelling results indicating a significant association between elevated levels of the comprehensive OC mixture in blood and an augmented risk of CVD. The OCPs exhibited the most substantial contribution to the observed association of OC mixture with CVD, particularly the pesticides β-HCH and transnonachlor. While the clearest exposure-response association was observed for the composite CVD, associations were also observed for MI and stroke.

The mixture assessment should be enhanced over specific-compound analysis, where the correlation between compounds affect the associations of specific compounds in isolation (copollutant confounding). However, when examining the compound within the context of a mixture, where correlations between compounds are assumed, a comprehensive understanding of its contribution to the overall effect size emerges. As a consequence, it is plausible that an individual compound analyses reveal significant associations, while their contribution within the mixture is inconsequential or even demonstrates a divergent direction of association.

The previous evidence on OC biomarkers in relation to cardiovascular end points is limited, although it is in concurrence with our current findings. In the preceding Prospective Investigation of the Vasculature in Uppsala Seniors (PIVUS) study^23^ including 898 participants, with blood concentrations of OCs highly comparable with those observed in this investigation, the adjusted ORs in the highest vs lowest quartile of incident ischemic stroke was 2.1 (95% CI, 0.7-6.2; *P* for trend = .11) for grouped total PCBs and 3.0 (95% CI, 1.0-9.4; *P* for trend = .03) for grouped OCPs (only including p,p′-DDE, transnonachlor, and HCB). Total OCs exposure was not measured but was included in a later cross-sectional assessment of the same participants where total OCs was associated with atherosclerotic plaques and echogenicity of the intima-media complex, suggesting that OCs might be involved in the early lipid infiltration of the vascular wall.^38^

In the Korean Cancer Prevention Study-II, including 526 participants and 111 patients with stroke, the hazard ratios in the third vs first tertile for incident stroke were 4.10 (95% CI, 1.58-10.6) for p,p′-DDE and similar for PCB-118, PCB-156, and PCB-138.^24^ Significant associations of OCPs and DLPCBs, but not total PCBs, with stroke were also seen (hazard ratio for third vs first tertile: OCP, 3.15; 95% CI, 1.30-7.65; *P* for trend = .008; total PCBs, 1.66; 95% CI, 0.80-3.45; *P* for trend = .13; DL PCBs, 3.17; 95% CI, 1.29-7.77; *P* for trend < .001). Separate analyses conducted by type of stroke suggested a stronger link with ischemic stroke, but caution is warranted since only few patients with hemorrhagic stroke were involved.^24^ In the cross-sectional National Health and Nutrition Examination Survey 1999-2002 study including 889 adults and 108 participants with self-reported CVD, DL PCBs, non-DL PCBs, and OCPs were significantly associated with prevalent CVD only among women. Compared with those in the lowest quartile of serum concentration, the OR for CVD across increasing quartiles were 0.9 (95% CI, 0.2-3.5), 2.0 (95% CI, 0.5-7.6), and 5.0 (95% CI, 1.2-20.4) for DL PCBs (*P* for trend < .01); 1.2 (95% CI, 0.4-4.0), 1.2 (95% CI, 0.4-4.2), and 3.8 (95% CI, 1.1-12.8) for non-DL PCBs (*P* for trend < .01); and 1.9 (95% CI, 0.5-7.7), 1.7 (95% CI, 0.4-7.1), and 4.0 (95% CI, 1.0-17.1) for OCPs (*P* for trend = .03).^39^ Finally, an OR of 1.59 (95% CI, 1.12-2.26) for CVD prescribed drugs comparing extreme quartiles of adipose tissue OCs mixture was reported, using an approach (weighted quantile sum), which, unlike quantile g-computation, only enables the simultaneous evaluation of compounds exhibiting the same directional effect (ie, β-HCH, p,p′-DDE, HCB, PCB-138, PCB-153, and PCB-180).^40^ Given that all the studies mentioned expressed contaminants as nanograms per gram of lipids, a direct comparison with our concentrations cannot be conducted. However, it should be noted that the same contaminants at higher and lower levels were consistently detected in all studies.

Exposure to OCs might contribute to CVD risk through multiple mechanisms. Extensive evidence from experimental studies has shown that exposure to PCBs directly impairs endothelial function^41,42,43^ and induces the formation of atherosclerotic plaques via inflammation,^9,44^ oxidative stress,^45,46^ immunotoxicity-related mechanisms,^47^ and by altering gene expression patterns in vascular cells.^48^ OCPs can induce endothelial dysfunction through increased reactive oxygen species generation via nicotinamide adenine dinucleotide phosphate oxidase expression and reduced bioavailability of nitric oxide.^46^ Exposure to a low concentration of OCP mixtures impaired glucose metabolism and mitochondrial function in L6 myotubes and zebrafish.^49,50^ In our study, β-HCH and transnonachlor demonstrated the highest individual influence on the OC joint mixture effect size. β-HCH exhibits characteristics of both an endocrine disrupting chemical and an activator of AhR signaling, thereby facilitating the development of oxidative stress, as well as has the capacity to cause DNA damage through H2AX phosphorylation.^51^ Similarly, elevated levels of transnonachlor have been linked to clinical manifestations of atherosclerosis,^52^ compromised glycemic control, and impaired beta cell function.^53^ Exposure to OC mixtures in mice has also shown to be able to intensify oxidative and inflammatory stressors in the heart to overwhelm protective mechanisms allowing for adverse cardiac remodeling.^54^

Furthermore, both PCBs and OCPs accumulate in human adipose tissue,^55^ affecting its function and fat distribution, and could thereby represent a mechanistic link between adipose tissue inflammation and dysfunction and cardiometabolic diseases. Accumulation of OCs in human adipose tissue has been correlated with adipose tissue macrophage infiltration, adipocyte size, parameters of glucose metabolism, inflammation, and variation in fat distribution.^55,56^

The observed increased risk of CVD due to overall OC mixture exposure may have large population health impact, since the burden of CVD worldwide is very high and the exposure to OCs is still ubiquitous. Consequently, our findings call for increased awareness in the contributing role of dietary exposure to chemical pollutants in CVD etiology. Importantly, in many populations, fish is the main source of OC exposure,^14,19,20,21,22^ and the beneficial effects of fish consumption, especially attributed to the long-chain n-3 polyunsaturated fatty acids, may be diminished by coexposure to the OCs.^22^ This is a feasible explanation for the observed U-shaped associations between fish consumption and both CVD and mortality in Western countries.^57,58,59^

### Strengths and Limitations

Among the strengths, the present study is, to our knowledge, the first to implement innovative methods for environmental mixtures in epidemiology, reflecting combined effect size of the studied compounds, to estimate the associations of OC mixtures with CVD. We used quantile g-computation models that account for the cooccurrence of multiple components present in the real world as a complex mixture. As this approach provides a more realistic estimate of risk compared with the use of individual OCs, a more solid basis for risk assessments is obtained, which should also be more meaningful in a legislative context. However, within this method, we did not further investigate nonlinearity, nonadditivity, or interactions. Finally, it is particularly noteworthy that the study was based on a fairly large sample size with MI and ischemic stroke ascertainments via linkage to Swedish registers.

This study has limitations. In this study, we only used a baseline single measurement of contaminants in blood. However, OCs are characterized as hydrophobic molecules with high fat solubility, leading to their accumulation predominantly in adipose tissues, while maintaining a dynamic equilibrium with blood levels. Thus, OCs exhibit extended half-lives within the body, allowing them to persist over extended periods.^60^ Further, the intraclass correlations of OCs in blood sampled 10 years apart was high (approximately 0.8).^61^ Therefore, despite their environmental levels decreasing, we can assume that the baseline exposure categorization is maintained over the years. During fasting conditions, the equilibrium between the concentrations of OCs in blood and adipose tissue is maintained. Nevertheless, physiological alterations, such as weight loss, can increase the release of these compounds from adipose tissue into the bloodstream.^62^ In the absence of complete information on weight changes preceding recruitment and during follow-up, it is not possible to completely exclude the potential for exposure misclassification or confounding.

Because lipophilic chemicals are transported in lipoproteins,^63^ the concentrations of these chemicals have sometimes been normalized for total blood lipids. Yet given the differences in magnitude, small errors in the measurement of lipids can greatly distort the measurements of OCs. In addition, since OCs may alter lipid levels,^18^ correcting lipid-soluble compounds for lipids might bias estimates.^64^ This is particularly important in the case of potential lipid-mediated health outcomes, such as CVD, for which alterations in lipids could be within the causal pathway between the exposure and the disease. Furthermore, in this study, lipid levels were not highly correlated with OC levels (eTable 5 in Supplement 1). Given blood samples were taken fasting (and therefore unaffected by ingested fat) and in accordance with the aforementioned reasoning, we made the decision to refrain from standardizing the OC concentrations based on lipid levels. Instead, we opted to include lipid adjustments in the regression models as an additional covariate.^65^ Although the available high-quality data on diet, anthropometric measurements, and clinical parameters allowed us to control for important potential confounders, we cannot discard the possibility of residual or unmeasured confounding. Additionally, it is important to note that quantile g-computation does not facilitate the estimation of associations across different quantiles of the joint exposure mixture. This approach can only reveal a coefficient representing the odds of experiencing the outcome resulting from a simultaneous increment of all exposures by a single quantile, assuming linearity and additivity.

## Conclusions

In this prospective nested case-control study, we found that the higher the blood levels of overall OC mixture, the higher likelihood of having an MI or an ischemic stroke. Our findings indicate that low but continual exposure to OCs over the years—as it occurs in the current general population—might contribute to an increased risk to experience CVD, the major causes of death worldwide. While measures may be needed to reduce this exposure, it is imperative to replicate these findings in diverse settings, particularly through larger prospective cohort studies, to establish their robustness and generalizability.

## Notes

Supplement 1. **eMethods.** Further Information on Baseline Measurements of Organochlorine Compounds (OCs) **eTable 1.** Limits of Detection and Quantification and Percentage of Samples Below These Limits **eTable 2.** Multivariable-Adjusted Associations Between Plasma Biomarkers of Total Organochlorine Pesticides (OCPs) and Dioxin (DL) and Non-Dioxinlike (NDL) Polychlorinated Biphenyls (PCBs) and Risk of Cardiovascular Disease (CVD) Accounting for Potential Intermediate Cardiometabolic Factors in 1528 Men and Women From Two Pooled Cohorts **eTable 3.** Multivariable-Adjusted Associations Between Plasma Biomarkers of the Total Organochlorine Pesticides (OCPs) and Dioxin (DL) and Non-Dioxinlike (NDL) Polychlorinated Biphenyls (PCBs) and Risk of Composite Cardiovascular Disease (CVD) by Cohort **eTable 4.** Multivariable-Adjusted Associations Between Plasma Biomarkers of Single Polychlorinated Biphenyls (PCBs) and Risk of Composite Cardiovascular Disease (CVD) in 1528 Men and Women From 2 Pooled Cohorts **eTable 5.** Spearman Correlations Between Levels Grouped OC and Lipid Levels **eFigure 1.** Flowchart of the Prospective Nested Case-Control Design Using 2 Pooled Swedish Cohorts, the Swedish Mammography Cohort-Clinical (SMC-C) and the 60-Year-Olds Cohort (60YO) **eFigure 2.** Box Plots of Individual Plasma Concentrations of the Detected Organochlorine Compounds (OCs) in Men and Women From the 2 Pooled Cohorts **eFigure 3.** Spearman Correlations Between Single Plasma Concentrations of All Detected Organochlorine Compounds (OCs) in Men and Women From 2 Pooled Cohorts

Supplement 2.
Data Sharing Statement
