# Supplementary material for: Blood Levels of Organochlorine Contaminants Mixtures and Cardiovascular Disease
Source: JAMA Netw Open. 2023 Sep 12;6(9):e2333347. doi: 10.1001/jamanetworkopen.2023.33347 (PMC10498337; doi:10.1001/jamanetworkopen.2023.33347)
Supplement: Supplement 1. — eMethods. Further Information on Baseline Measurements of Organochlorine Compounds (OCs) eTable 1. Limits of Detection and Quantification and Percentage of Samples Below These Limits eTable 2. Multivariable-Adjusted Associations Between Plasma Biomarkers of Total Organochlorine Pesticides (OCPs) and Dioxin (DL) and Non-Dioxinlike (NDL) Polychlorinated Biphenyls (PCBs) and Risk of Cardiovascular Disease (CVD) Accounting for Potential Intermediate Cardiometabolic Factors in 1528 Men and Women From Two Pooled Cohorts eTable 3. Multivariable-Adjusted Associations Between Plasma Biomarkers of the Total Organochlorine Pesticides (OCPs) and Dioxin (DL) and Non-Dioxinlike (NDL) Polychlorinated Biphenyls (PCBs) and Risk of Composite Cardiovascular Disease (CVD) by Cohort eTable 4. Multivariable-Adjusted Associations Between Plasma Biomarkers of Single Polychlorinated Biphenyls (PCBs) and Risk of Composite Cardiovascular Disease (CVD) in 1528 Men and Women From 2 Pooled Cohorts eTable 5. Spearman Correlations Between Levels Grouped OC and Lipid Levels eFigure 1. Flowchart of the Prospective Nested Case-Control Design Using 2 Pooled Swedish Cohorts, the Swedish Mammography Cohort-Clinical (SMC-C) and the 60-Year-Olds Cohort (60YO) eFigure 2. Box Plots of Individual Plasma Concentrations of the Detected Organochlorine Compounds (OCs) in Men and Women From the 2 Pooled Cohorts eFigure 3. Spearman Correlations Between Single Plasma Concentrations of All Detected Organochlorine Compounds (OCs) in Men and Women From 2 Pooled Cohorts [file jamanetwopen-e2333347-s001.pdf]

Supplemental Online Content

Donat-Vargas C, Schillemans T, Kiviranta H, et al. Blood levels of organochlorine contaminants mixtures and cardiovascular disease. *JAMA Netw Open*. 2023;6(9):e2333347

eMethods. Further Information on Baseline Measurements of Organochlorine Compounds (OCs)

eTable 1. Limits of Detection and Quantification and Percentage of Samples Below These Limits

eTable 2. Multivariable-Adjusted Associations Between Plasma Biomarkers of Total Organochlorine Pesticides (OCPs) and Dioxin (DL) and Non-Dioxinlike (NDL) Polychlorinated Biphenyls (PCBs) and Risk of Cardiovascular Disease (CVD) Accounting for Potential Intermediate Cardiometabolic Factors in 1528 Men and Women From Two Pooled Cohorts

eTable 3. Multivariable-Adjusted Associations Between Plasma Biomarkers of the Total Organochlorine Pesticides (OCPs) and Dioxin (DL) and Non-Dioxinlike (NDL) Polychlorinated Biphenyls (PCBs) and Risk of Composite Cardiovascular Disease (CVD) by Cohort

eTable 4. Multivariable-Adjusted Associations Between Plasma Biomarkers of Single Polychlorinated Biphenyls (PCBs) and Risk of Composite Cardiovascular Disease (CVD) in 1528 Men and Women From 2 Pooled Cohorts

eTable 5. Spearman Correlations Between Levels Grouped OC and Lipid Levels

eFigure 1. Flowchart of the Prospective Nested Case-Control Design Using 2 Pooled Swedish Cohorts, the Swedish Mammography Cohort-Clinical (SMC-C) and the 60-Year-Olds Cohort (60YO)

eFigure 2. Box Plots of Individual Plasma Concentrations of the Detected Organochlorine Compounds (OCs) in Men and Women From the 2 Pooled Cohorts

eFigure 3. Spearman Correlations Between Single Plasma Concentrations of All Detected Organochlorine Compounds (OCs) in Men and Women From 2 Pooled Cohorts

This supplementary material has been provided by the authors to give readers additional information about their work.

© 2023 Donat-Vargas C et al. *JAMA Network Open*.

**eMethods.** Further Information on Baseline Measurements of Organochlorine Compounds (OCs)
Organochlorine compounds (OCs) were measured at the Finnish Institute for Health and Welfare by gas chromatography-triple quadrupole mass spectrometry (GC–MS/MS).<br>Limits of detection (LOD) ranged from 2 pg/mL for PCBs and trans-nonachlor to 16 pg/mL for p,p'-DDE and PCB-28. Limits of quantification (LOQ) ranged from 5 pg/mL for the majority of PCBs and trans-nonachlor to 40 pg/mL for p,p'-DDE. Concentrations <LOQ were assigned the mid-value between LOD and LOQ.<br>The following seven compounds PCB-28, α-HCH, γ-HCH, PeCB and PBDEs (BDE-47, -153, -99) were not further considered in the analyzes because >75% of the samples were < LOQ.<br>We analyzed individual and grouped OCs. Organochlorine pesticides (OCPs) included p,p'-DDE, p,p'-DDT, HCB, β-HCH, trans-nonachlor and oxychlordane. PCBs congeners were classified into dioxin-like (DL)-PCBs and non-dioxin-like (NDL)-PCBs. DL-PCBs included the congeners PCB-118 and 156, and NDL-PCBs included all other PCBs congeners, 52, 74, 99, 101, 138, 153, 170, 180, 183 and 187. Total PCBs included DL-PCBs and NDL-PCBs<br>Since the toxicity varies between the compounds, we used standardized-based summary measures to prevent the sum from being determined solely by the compounds in higher concentrations. Thus, each compound was first rescaled to mean = 0 and standard deviation (SD) = 1 and then compounds were summed per group (DL-PCBs, NDL-PCBs, and OCPs)

© 2023 Donat-Vargas C et al. *JAMA Network Open*.

eTable 1. Limits of Detection and Quantification and Percentage of Samples Below These Limits				
**Compound**	**LOD (pg/mL)**	**LOQ (pg/mL)**	**% <LOD**	**% <LOQ**
PCB 28	16	40	56.5	80.2
PCB 52	2	5	24.0	58.0
PCB 74	2	5	0	0.07
PCB 99	2	5	0.26	2.03
PCB 101	2	5	9.2	46.5
PCB 118	2	5	0	0
PCB 138	2	5	0	0
PCB 153	2	5	0	0
PCB 156	2	5	0.07	0.07
PCB 170	2	5	0	0
PCB 180	2	5	0	0
PCB 187	2	5	0	0.07
PCB 183	2	5	0.20	0.52
p,p'-DDE	16	40	0	0.13
p,p'-DDT	6	15	12.0	21.3
HCB	4	10	0	0
α-HCH	8	20	90.8	99.2
β-HCH	6	15	0	0.46
γ-HCH	8	20	94.7	98.4
Transnonachlor	2	5	0	0.07
Oxychlordane	10	25	0.39	10.3
PeCB	4	10	61.9	99.0
BDE 47	6	15	45.6	76.4
BDE 153	6	15	76.5	96.3
BDE 99	6	15	80.2	94.0

BDE= brominated diphenyl ether**; p,p'-DDE**=dichlorodiphenyldichloroethene; **p,p'-DDT**= dichlorodiphenyltrichloroethane; **HCB**= hexachlorobenzene; **HCH**= hexachlorocyclohexane; **LOD**= limits of detection ; **LOQ**= limits of quantification **PeCB**= pentachlorobenzene; **PCBs**= polychlorinated biphenyls. Because of low prevalence of detected concentrations of **PCB-28**, α-**HCH,** γ**-HCH, PeCB** and the **PBDEs**, these compounds were not included in any analyses

© 2023 Donat-Vargas C et al. *JAMA Network Open*.

eTable 2. Multivariable-Adjusted Associations Between Plasma Biomarkers of Total Organochlorine Pesticides (OCPs) and Dioxin (DL) and Non-Dioxinlike (NDL) Polychlorinated Biphenyls (PCBs) and Risk of Cardiovascular Disease (CVD) Accounting for Potential Intermediate Cardiometabolic Factors in 1528 Men and Women From Two Pooled Cohorts

		Composite CVD risk		
	**Control/Case**	**OR (95%CI)[1]**	**OR (95%CI)[2]**	**OR (95%CI)[3]**
**Total OCPs**				
Quartile 1	208/137	1 (Ref.)	1 (Ref.)	1 (Ref.)
Quartile 2	207/168	1.13 (0.80, 1.59)	1.13 (0.80, 1.60)	1.12 (0.79, 1.59)
Quartile 3	207/171	1.16 (0.81, 1.67)	1.17 (0.82, 1.69)	1.16 (0.80, 1.66)
Quartile 4	207/223	1.47 (1.02, 2.14)	1.45 (1.00, 2.12)	1.42 (0.97, 2.07)
P trend		0.03	0.04	0.06
**DL-PCBs**				
Quartile 1	208/150	1 (Ref.)	1 (Ref.)	1 (Ref.)
Quartile 2	207/174	1.10 (0.79-1.53)	1.11 (0.80, 1.54)	1.11 (0.80, 1.55)
Quartile 3	207/190	1.21 (0.86, 1.69)	1.21 (0.86, 1.70)	1.20 (0.85, 1.69)
Quartile 4	207/185	1.15 (0.80, 1.66)	1.13 (0.78, 1.63)	1.13 (0.78, 1.63)
P trend		0.45	0.54	0.56
**NDL-PCBs**				
Quartile 1	208/154	1 (Ref.)	1 (Ref.)	1 (Ref.)
Quartile 2	207/167	1.04 (0.76, 1.44)	1.04 (0.76, 1.44)	1.06 (0.76, 1.46)
Quartile 3	207/198	1.10 (0.79, 1.54)	1.11 (0.80, 1.55)	1.12 (0.80, 1.56)
Quartile 4	207/180	0.99 (0.70, 1.42)	0.99 (0.69, 1.41)	0.99 (0.69, 1.42)
P trend		0.93	0.92	0.92

Cohort-specific quartiles based on controls.
To create the sum variables, each of the compounds was rescaled to mean = 0 and standard deviation (SD) = 1 before all being added.

Organochlorine pesticides include the most prevalent ones: pp-DDE, HCB, β-HCH, transnonachlor, oxychlordane and pp-DDT. DL- PCBs (Dioxin-like-PCBs) include PCB118 and 156. NDL- PCBs (Non-dioxin-like-PCBs) include PCB28, 52, 74, 99, 10, 138, 153, 170, 180, 183 and 187

1 Model adjusted for matching factors (sex, age, sampling date and cohort) and education, physical activity, smoking status, healthy diet score, fish consumption, BMI, family history of myocardial infraction plus total cholesterol and triglycerides blood levels.
2 Model 1 further adjusted for hypertension
3 Model 2 further adjusted for diabetes

© 2023 Donat-Vargas C et al. *JAMA Network Open*.

eTable 3. Multivariable-Adjusted Associations Between Plasma Biomarkers of the Total Organochlorine Pesticides (OCPs) and Dioxin (DL) and Non-Dioxinlike (NDL) Polychlorinated Biphenyls (PCBs) and Risk of Composite Cardiovascular Disease (CVD) by Cohort

	SMC-C cohort (*n*=742)			60YO cohort (*n*=786)		
	**Co./Ca.**	**OR (95%CI)[1]**	**OR (95%CI)[2]**	**Co./Ca.**	**OR (95%CI)[1]**	**OR (95%CI)[2]**
**Total OCPs**						
Quartile 1	109/54	1 (Ref.)	1 (Ref.)	99/83	1 (Ref.)	1 (Ref.)
Quartile 2	109/74	1.48 (0.93-2.36)	1.38 (0.84-2.27)	98/94	1.12 (0.75-1.69)	1.04 (0.65-1.65)
Quartile 3	109/84	1.69 (1.04-2.73)	1.41 (0.84-2.37)	98/87	1.04 (0.69-1.59)	1.08 (0.68-1.73)
Quartile 4	109/94	1.93 (1.17-3.18)	1.67 (0.99-2.84)	98/129	1.58 (1.06-2.34)	1.65 (1.04-2.60)
P trend		0.02	0.09		0.02	0.02
**DL-PCBs**						
Quartile 1	109/63	1 (Ref.)	1 (Ref.)	99/87	1 (Ref.)	1 (Ref.)
Quartile 2	109/82	1.37 (0.86- 2.17)	1.32 (0.81-2.14)	98/92	1.07 (0.73-1.59)	0.99 (0.64-1.54)
Quartile 3	109/85	1.33 (0.83-2.12)	1.35 (0.82-2.22)	98/105	1.22 (0.82-1.83)	1.23 (0.78-1.93)
Quartile 4	109/76	1.22 (0.74-2.01)	1.17 (0.69-1.99)	98/109	1.28 (0.85-1.91)	1.38 (0.88-2.18)
P trend		0.67	0.76		0.20	0.10
**NDL-PCBs**						
Quartile 1	109/67	1 (Ref.)	1 (Ref.)	99/87	1 (Ref.)	1 (Ref.)
Quartile 2	109/82	1.23 (0.80-1.89)	1.15 (0.73-1.79)	98/85	0.98 (0.66-1.47)	0.99 (0.63-1.55)
Quartile 3	109/94	1.40 (0.89-2.19)	1.23 (0.77-1.98)	98/104	1.22 (0.82-1.82)	1.15 (0.73-1.80)
Quartile 4	109/63	0.89 (0.54-1.47)	0.80 (0.47-1.36)	98/117	1.38 (0.93-2.05)	1.46 (0.94-2.27)
P trend		0.52	0.31		0.07	0.06

Cohort-specific quartiles based on controls.
To create the sum variables, each of the compounds was rescaled to mean = 0 and standard deviation (SD) = 1 before all being added.

Organochlorine pesticides include pp-DDE, pp-DDT, HCB, β-HCH, transnonachlor and oxychlordane. DL- PCBs (Dioxin-like-PCBs) include PCB-118 and 156. NDL- PCBs (Non-dioxin-like-PCBs) include PCB-28, 52, 74, 99, 10, 138, 153, 170, 180, 183 and 187.
1 Model adjusted for matching factors: sex, age, sampling date.
2 Model further adjusted for education, physical activity, smoking status, healthy diet score, fish consumption, BMI, and family history of myocardial infraction.

© 2023 Donat-Vargas C et al. *JAMA Network Open*.

eTable 4. Multivariable-Adjusted Associations Between Plasma Biomarkers of Single Polychlorinated Biphenyls (PCBs) and Risk of Composite Cardiovascular Disease (CVD) in 1528 Men and Women From 2 Pooled Cohorts			
		**Composite CVD risk**	
	**Control/Case.**	**OR (95%CI)[1]**	**OR (95%CI)[2]**
**PCB 52**			
<LOQ	470/416	1 (Ref.)	1 (Ref.)
>LOQ	359/283	1.23 (0.91-1.67)	1.21 (0.88-1.67)
**PCB 74**			
Tertile 1	280/191	1 (Ref.)	1 (Ref.)
Tertile 2	275/267	1.49 (1.15-1.94)	1.51 (1.14-2.01)
Tertile 3	274/241	1.29 (0.98-1.71)	1.31 (0.97-1.77)
P trend		0.15	0.19
**PCB 99**			
Tertile 1	284/220	1 (Ref.)	1 (Ref.)
Tertile 2	269/240	1.15 (0.89-1.48)	1.03 (0.78-1.37)
Tertile 3	276/239	1.08 (0.83-1.40)	1.02 (0.77-1.35)
P trend		0.66	0.93
**PCB 101**			
<LOQ	382/328	1 (Ref.)	1 (Ref.)
>LOQ	447/371	0.97 (0.79-1.20)	1.10 (0.88-1.38)
**PCB 118**			
Tertile 1	277/226	1 (Ref.)	1 (Ref.)
Tertile 2	277/246	1.09 (0.84-1.41)	1.21 (0.91-1.60)
Tertile 3	275/227	0.99 (0.76-1.28)	1.13 (0.85-1.51)
P trend		0.83	0.51
**PCB 138**			
Tertile 1	277/208	1 (Ref.)	1 (Ref.)
Tertile 2	276/243	1.19 (0.92-1.53)	1.08 (0.83-1.42)
Tertile 3	276/248	1.19 (0.92-1.54)	1.08 (0.82-1.43)
P trend		0.24	0.62
**PCB 153**			
Tertile 1	277/198	1 (Ref.)	1 (Ref.)
Tertile 2	277/261	1.33 (1.03-1.71)	1.25 (0.95-1.64)
Tertile 3	275/240	1.23 (0.94-1.61)	1.16 (0.87-1.54)
P trend		0.21	0.44
**PCB 156**			
Tertile 1	277/213	1 (Ref.)	1 (Ref.)
Tertile 2	278/222	1.05 (0.80-1.37)	0.92 (0.69-1.23)
Tertile 3	274/264	1.28 (0.97-1.68)	1.18 (0.88-1.58)
P trend		0.06	0.16
**PCB 170**			
Tertile 1	281/209	1 (Ref.)	1 (Ref.)
Tertile 2	273/228	1.11 (0.86-1.44)	1.05 (0.80-1.39)
Tertile 3	275/262	1.29 (0.99-1.69)	1.20 (0.90-1.59)
P trend		0.05	0.20
**PCB 180**			
Tertile 1	277/201	1 (Ref.)	1 (Ref.)
Tertile 2	279/239	1.19 (0.92-1.53)	1.11 (0.84-1.46)

© 2023 Donat-Vargas C et al. *JAMA Network Open*.

Tertile 3	273/259	1.33 (1.02-1.74)	1.26 (0.94-1.69)
P trend		0.04	0.12
**PCB 183**			
Tertile 1	277/204	1 (Ref.)	1 (Ref.)
Tertile 2	277/250	1.26 (0.98-1.62)	1.16 (0.88-1.53)
Tertile 3	275/245	1.20 (0.93-1.55)	1.08 (0.82-1.42)
P trend		0.25	0.73
**PCB 187**			
Tertile 1	280/206	1 (Ref.)	1 (Ref.)
Tertile 2	273/254	1.26 (0.98-1.62)	1.17 (0.89-1.54)
Tertile 3	276/239	1.19 (0.91-1.55)	1.08 (0.81-1.44)
P trend		0.27	0.68

Cohort-specific tertiles based on controls.
1 Model adjusted for matching factors: sex, age, sampling date and cohort.
2 Model further adjusted for education, physical activity, smoking status, healthy diet score, fish consumption, BMI, and family history of myocardial infraction.

© 2023 Donat-Vargas C et al. *JAMA Network Open*.

eTable 5. Spearman Correlations Between Levels Grouped OC and Lipid Levels

	Triglycerides	Total cholesterol
**Total PCBs**	0.29*	0.26*
**DL-PCBs**	0.27*	0.30*
**NDL-PCBs**	0.28*	0.25*
**OCPs**	0.37*	0.26*

*All correlations were statistically significant (p value< 0.05)

p,p'-DDE=dichlorodiphenyldichloroethene; p,p'-DDT= dichlorodiphenyltrichloroethane; HCB= hexachlorobenzene; HCH= hexachlorocyclohexane; PCBs= polychlorinated biphenyls;

Organochlorine pesticides (OCPs) include pp-DDE, pp-DDT, HCB, β-HCH, transnonachlor and oxychlordane.
Total PCBs include all PCBs congeners; DL- PCBs (Dioxin-like-PCBs) include PCB-118 and 156; NDL- PCBs (Non-dioxin-like-PCBs) include PCB-28, 52, 74, 99, 10, 138, 153, 170, 180, 183 and 187.

© 2023 Donat-Vargas C et al. *JAMA Network Open*.

eFigure 1. Flowchart of the Prospective Nested Case-Control Design Using 2 Pooled Swedish Cohorts, the Swedish Mammography Cohort-Clinical (SMC-C) and the 60-Year-Olds Cohort (60YO)

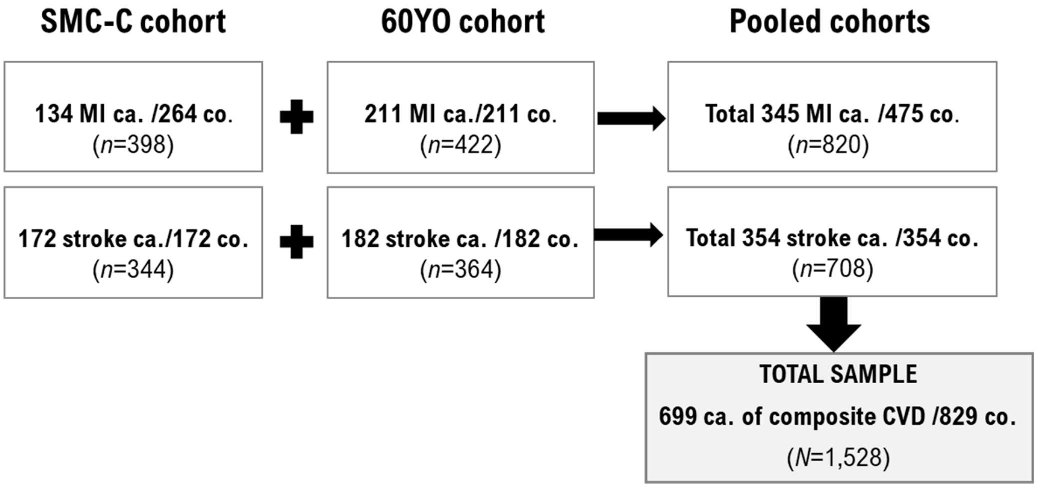

Abreviations:60YO= 60-year-olds cohort; ca.= case; co.=control; CVD=cardiovascular disease; MI=myocardial infarction; SMC-C=Swedish Mammography Cohort-Clinical cohort.

© 2023 Donat-Vargas C et al. *JAMA Network Open*.

eFigure 2. Box Plots of Individual Plasma Concentrations of the Detected Organochlorine Compounds (OCs) in Men and Women From the 2 Pooled Cohorts

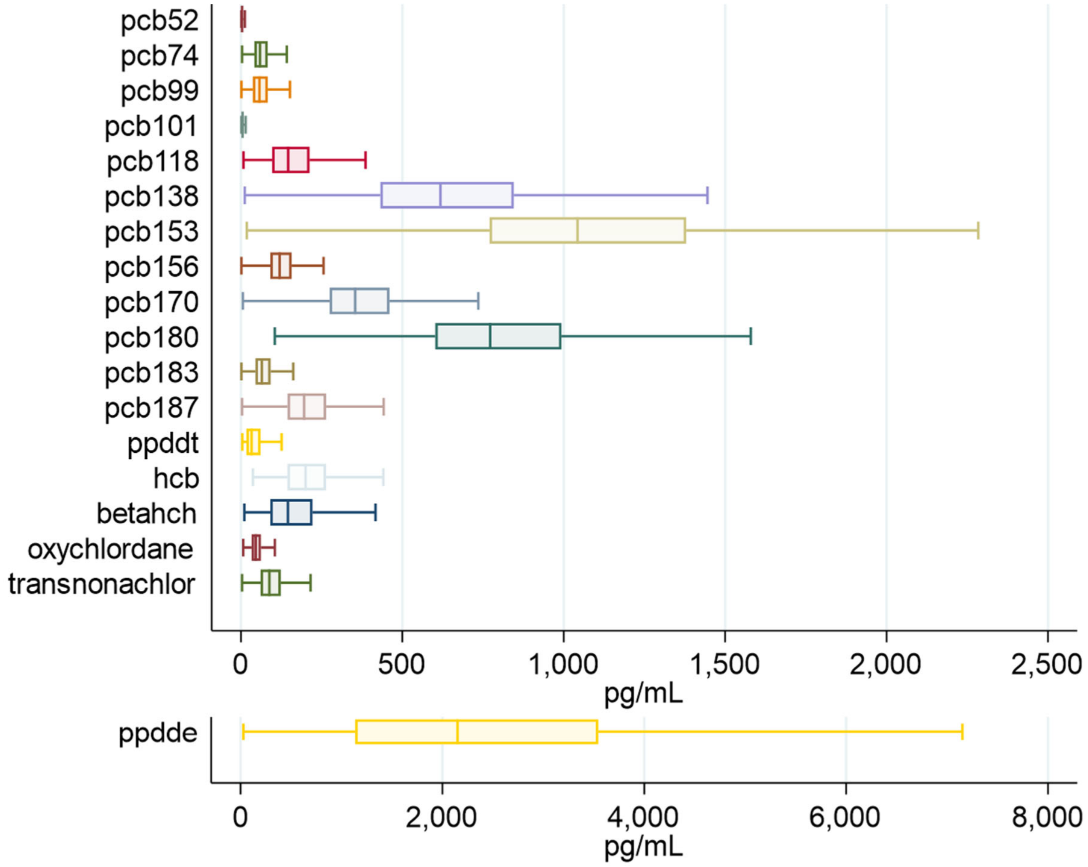

Box plots of each individual OC, display lower adjacent value, 25th percentile (lower hinge), median, 75th percentile (upper hinge) and the upper adjacent value

© 2023 Donat-Vargas C et al. *JAMA Network Open*.

eFigure 3. Spearman Correlations Between Single Plasma Concentrations of All Detected Organochlorine Compounds (OCs) in Men and Women From 2 Pooled Cohorts

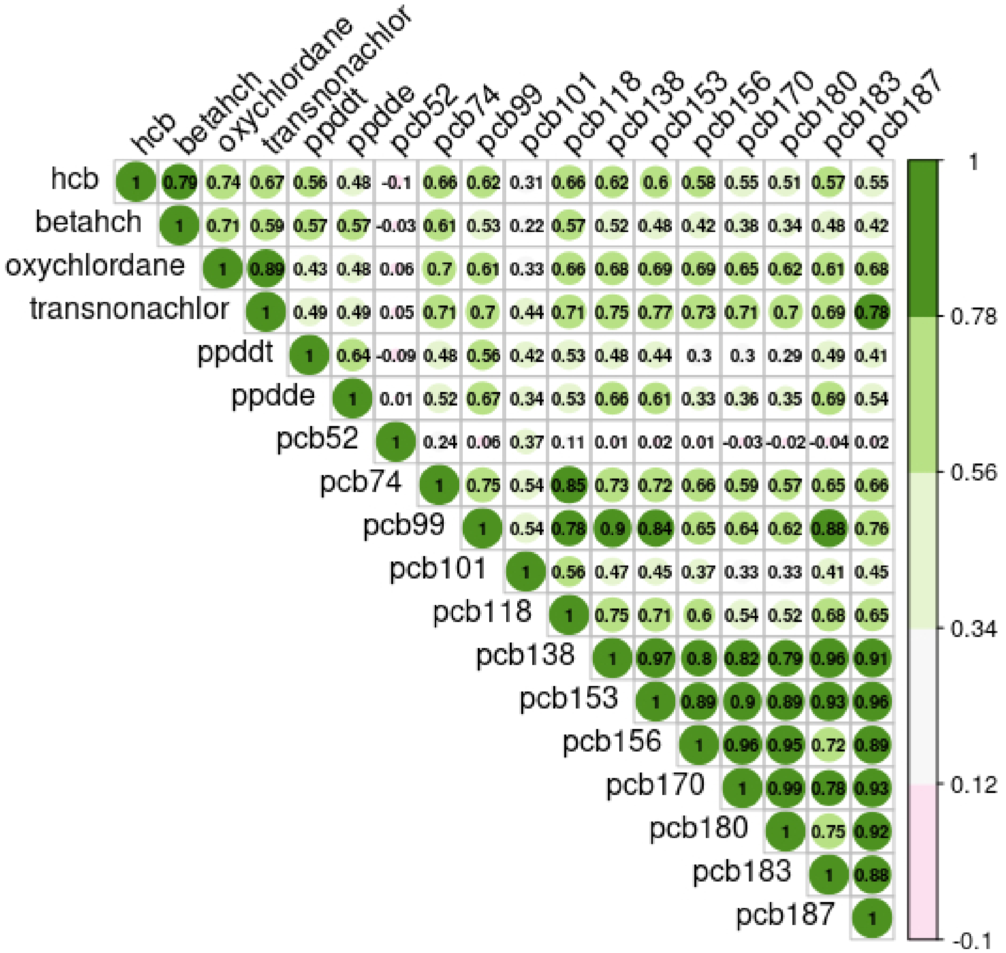

© 2023 Donat-Vargas C et al. *JAMA Network Open*.
