# Supplementary material for: Blood Levels of Organochlorine Contaminants Mixtures and Cardiovascular Disease
Source: JAMA Netw Open. 2023 Sep 12;6(9):e2333347. doi: 10.1001/jamanetworkopen.2023.33347 (PMC10498337; doi:10.1001/jamanetworkopen.2023.33347)
Supplement: Supplement 2. — Data Sharing Statement [file jamanetwopen-e2333347-s002.pdf]

Data Sharing Statement

Donat-Vargas. Blood Levels of Organochlorine Contaminants Mixtures and Cardiovascular Disease. *JAMA Netw Open*. Published September 12, 2023.
doi:10.1001/jamanetworkopen.2023.33347

Data
Data available: No
